# *Helicobacter pylori* infection prevalence declined among an urban health check-up population in Chengdu, China: a longitudinal analysis of multiple cross-sectional studies

**DOI:** 10.3389/fpubh.2023.1128765

**Published:** 2023-11-27

**Authors:** Jin-Chen Zou, Mao-Yao Wen, Yan Huang, Xin-Zu Chen, Jian-Kun Hu, on behalf of the SIGES Research Group

**Affiliations:** ^1^Gastric Cancer Center & Gastric Cancer Laboratory, Department of General Surgery, West China Hospital, Sichuan University, Chengdu, China; ^2^Department of General Surgery, Second People’s Hospital of Yibin City – West China Yibin Hospital, Sichuan University, Yibin, China; ^3^Health Management Center, General Practice Medical Center, West China Hospital, Sichuan University, Chengdu, China; ^4^Department of Gastrointestinal Surgery, People’s Hospital of Ya’an City – West China Ya’an Hospital, Sichuan University, Ya’an, China; ^5^Yibin Cancer Prevention and Control Center, Second People’s Hospital of Yibin City – West China Yibin Hospital, Sichuan University, Yibin, China

**Keywords:** gastric cancer, *Helicobacter pylori*, epidemiology, screening, health policy

## Abstract

**Objectives:**

The efficacy of updated health policy in improving the generalization of *Helicobacter pylori* screening and eradication in southwest China was assessed in a longitudinal analysis of multiple cross-sectional studies from an institution.

**Methods:**

In the periods 2009–2010, 2013–2014, and 2019–2021, 8,365, 16,914, and 18,281 urban observations from health check-ups at West China Hospital were analyzed, respectively. The ^14^C-urea or ^13^C-urea breath test was consistently used for *H. pylori* detection. The protocol has been reported elsewhere (PROSPERO Registration number: CRD42019120764).

**Results:**

The overall prevalence of *H. pylori* dramatically decreased from 53.1% to 30.7% over the past decade (OR = 0.39, 95% CI 0.37–0.41), with a similar decline in all sex-specific and age-specific subgroups. The age-specific prevalence consistently increased before 40 years of age and always peaked at 50–59 years. Longitudinal clearance increased along with aging, and prevalence dropped to 22.6%, 25.1%, and 23.6% in the 40–49, 50–59, and 60–69 years initial age groups, respectively.

**Conclusion:**

The generalization of *H. pylori* screening and eradication could greatly contribute to the control of *H. pylori* infection among urban health check-up populations and lower gastric cancer incidence.

## Introduction

The incidence of and mortality from gastric cancer has been high in China over recent decades (1). *H. pylori* is the major risk pathogen for gastric cancer in eastern and western populations (2, 3). A nationwide decision-making analysis in China suggested that *H. pylori* eradication is a cost-saving strategy, with general annual endoscopic screening dominating other protocols (4). Targeted and tailored endoscopic screening among high-risk subpopulations could be more cost-effective from a willingness-to-pay perspective (4). Additionally, it has been highlighted that active *H. pylori* eradication through the organized screening and surveillance of high-risk subpopulations should be a systematic measure for controlling gastric cancer-specific mortality, especially in high-risk areas. In China, extensive screening of *H. pylori* has been practiced more commonly in recent years along with updated health policy (5, 6), and infected individuals are consequently actively advised and treated for *H. pylori* eradication. Therefore, this study aimed to assess the efficacy of the updated health policy in improving and extending *H. pylori* screening and eradication in China.

## Methods

To evaluate the extensive screening and eradication of *H. pylori* in a macroscopic view, we longitudinally observed the prevalence of *H. pylori* in health check-ups over the past decade at the Health Management Center of West China Hospital, a central high-volume hospital in southwest China. The targeted populations included self-reported asymptomatic observations from health check-ups. The health check-ups were organized by certain public institutions or enterprises, as well as a few people from urban areas. The ^14^C-urea or ^13^C-urea breath test (UBT) was used for *H. pylori* detection.

Crude data were retrieved from three cross-sectional studies (two published and one unpublished) in the periods 2009–2010, 2013–2014, and 2019–2021, respectively (7, 8). The sex-specific and age-specific prevalence of *H. pylori* was calculated. Odds ratios (OR) and 95% confidence intervals (CI) were estimated where applicable. Additionally, *P*_trend_ values were tested over the decade. The longitudinal *H. pylori* infection clearance of each initial age group was estimated, i.e., the prevalence difference between the periods 2019–2021 and 2009–2010.

The protocol has been reported elsewhere (PROSPERO Registration Number: CRD42019120764) (9). The Sichuan Gastric Cancer Early Detection and Screening (SIGES) study was approved by the Biomedical Ethical Committee of West China Hospital, Sichuan University (id: 2015-151-V2; 2018-215-V1).

## Results

**Figure 1:**
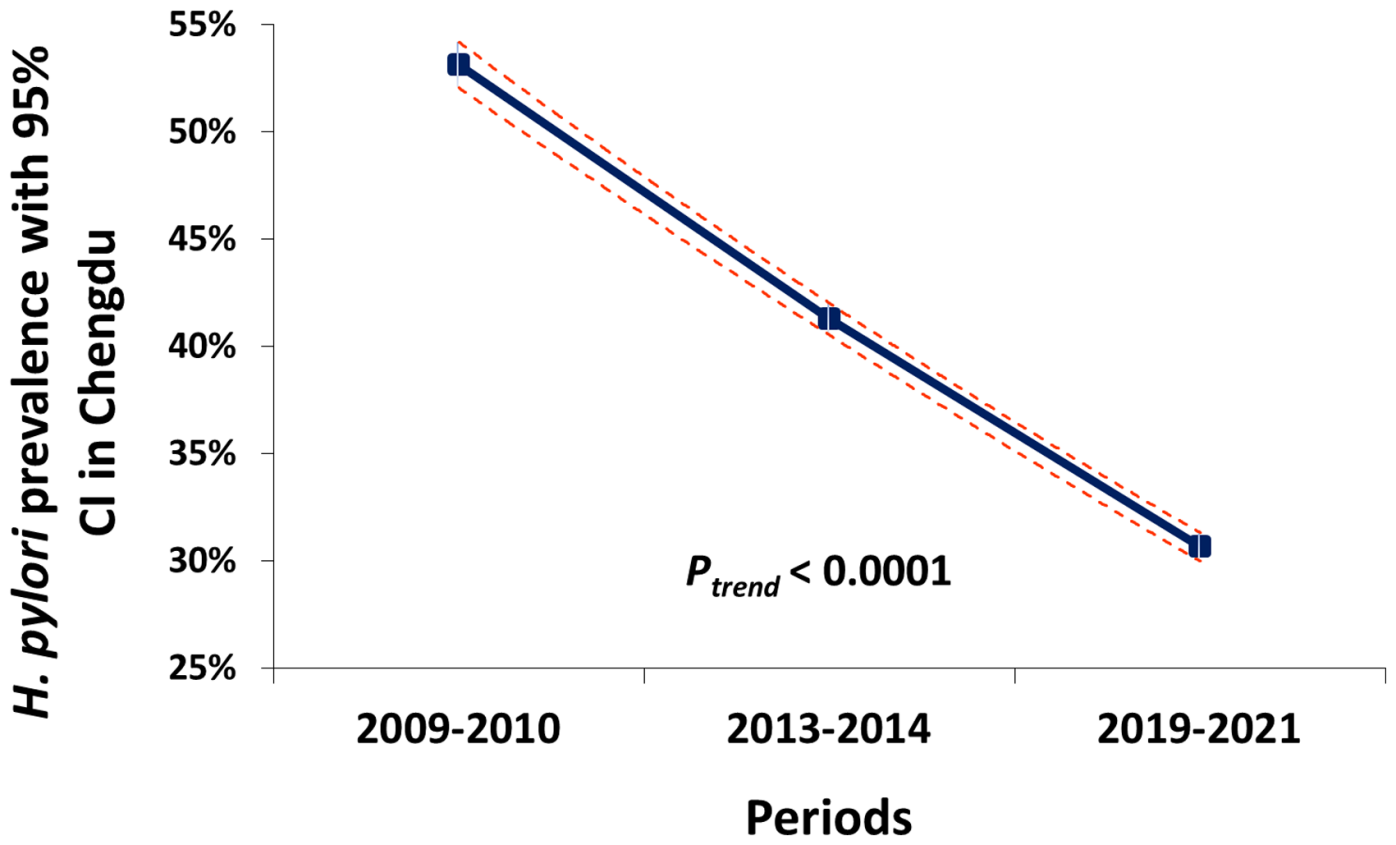
The prevalence trend of *H. pylori* infection among the urban health check-up population in Chengdu.

In the periods 2009–2010, 2013–2014, and 2019–2021, 8,365, 16,914, and 18,281 observations from health check-ups were analyzed, respectively. The overall prevalence of *H. pylori* dramatically decreased from 53.1% to 30.7% over the past decade among urban health check-up populations (OR = 0.39, 95% CI 0.37–0.41; *P*_trend_ < 0.0001) (Table 1; Figure 1).

**Table 1:** The 10-year trend of *H. pylori* infection prevalence among the urban health check-up population in Chengdu.

Subgroup	2009–2010			2013–2014				2019–2021				*P*_trend_
	Infected	Sum	Prevalence	Infected	Sum	Prevalence	OR (95% CI)^#^	Infected	Sum	Prevalence	OR (95% CI)^#^	
Total	4,444	8,365	53.1%	6,981	16,914	41.3%	0.62 (0.59–0.65)	5,605	18,281	30.7%	0.39 (0.37–0.41)	<0.0001
Sex												
Female	1,771	3,424	51.7%	3,640	8,879	41.0%	0.65 (0.60–0.70)	2,570	8,544	30.1%	0.40 (0.37–0.44)	<0.0001
Male	2,673	4,941	54.1%	3,341	8,035	41.6%	0.60 (0.56–0.65)	3,035	9,737	31.2%	0.38 (0.36–0.41)	<0.0001
Age												
<20 yrs	4	11	36.4%	124	389	31.9%	0.82 (0.24–2.85)	22	85	25.9%	0.61 (0.16–2.29)	0.0001
20–29 yrs	184	412	44.7%	637	1,570	40.6%	0.85 (0.68–1.05)	374	1,463	25.6%	0.43 (0.34–0.53)	<0.0001
30–39 yrs	1,016	2,030	50.0%	1,213	3,021	40.2%	0.67 (0.60–0.75)	1,131	3,843	29.4%	0.42 (0.37–0.47)	<0.0001
40–49 yrs	1,698	3,091	54.9%	2,074	4,863	42.6%	0.61 (0.56–0.67)	1,824	5,836	31.6%	0.37 (0.34–0.41)	<0.0001
50–59 yrs	972	1,714	56.7%	1,583	3,715	42.6%	0.57 (0.50–0.64)	1,543	4,773	32.3%	0.36 (0.33–0.41)	<0.0001
60–69 yrs	412	792	52.0%	937	2,329	40.2%	0.62 (0.53–0.73)	618	1,954	31.6%	0.43 (0.36–0.51)	<0.0001
≥70 yrs	158	315	50.2%	413	1,027	40.2%	0.67 (0.52–0.86)	93	327	28.4%	0.39 (0.28–0.55)	<0.0001

CI, confidence interval; *H. pylori*, *Helicobacter pylori*; OR, odds ratio; yrs, years. # refers to the period 2009–2010.

**Figure 2:**
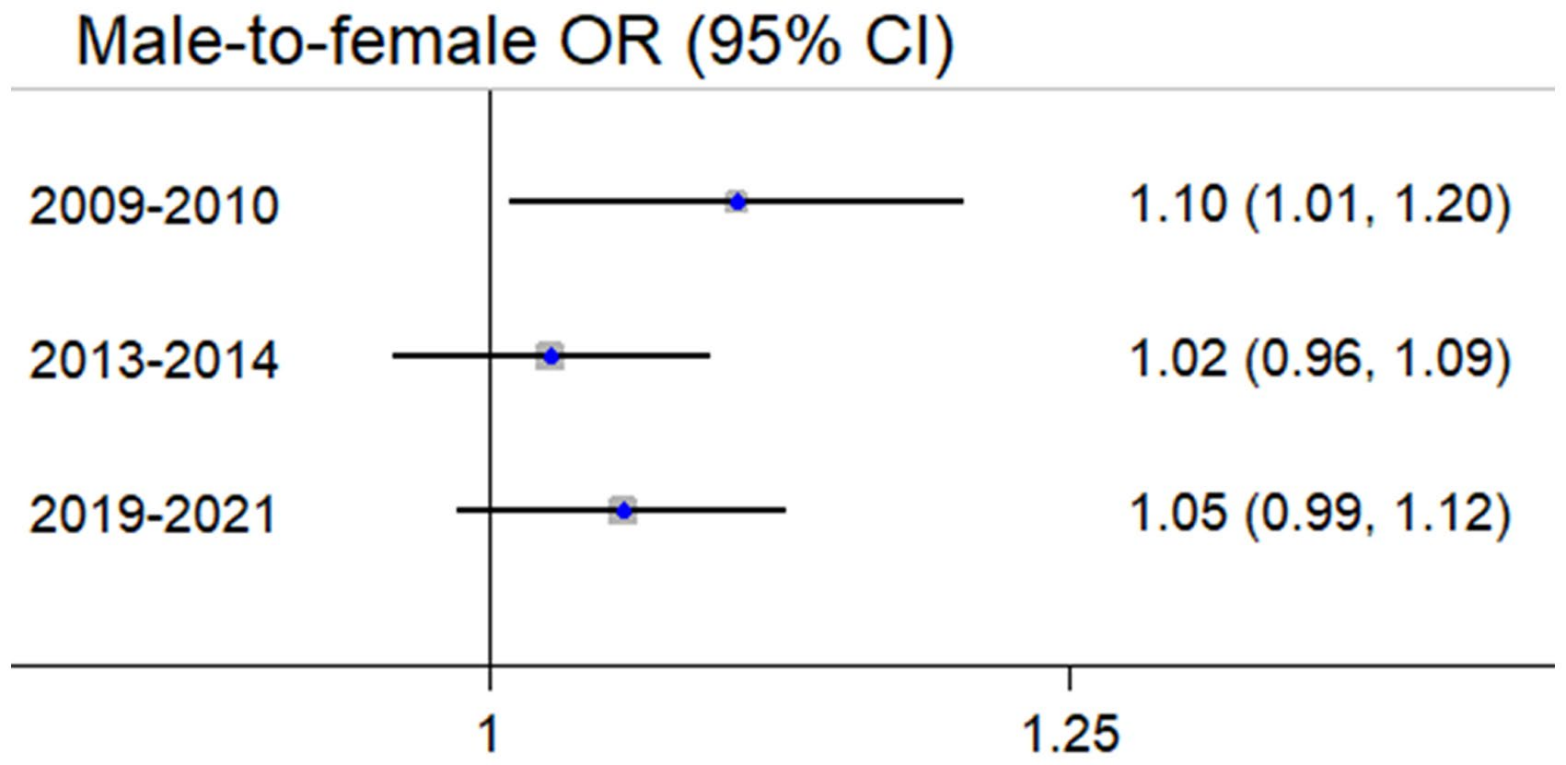
Sex-specific comparison of *H. pylori* infection prevalence.

**Figure 3:**
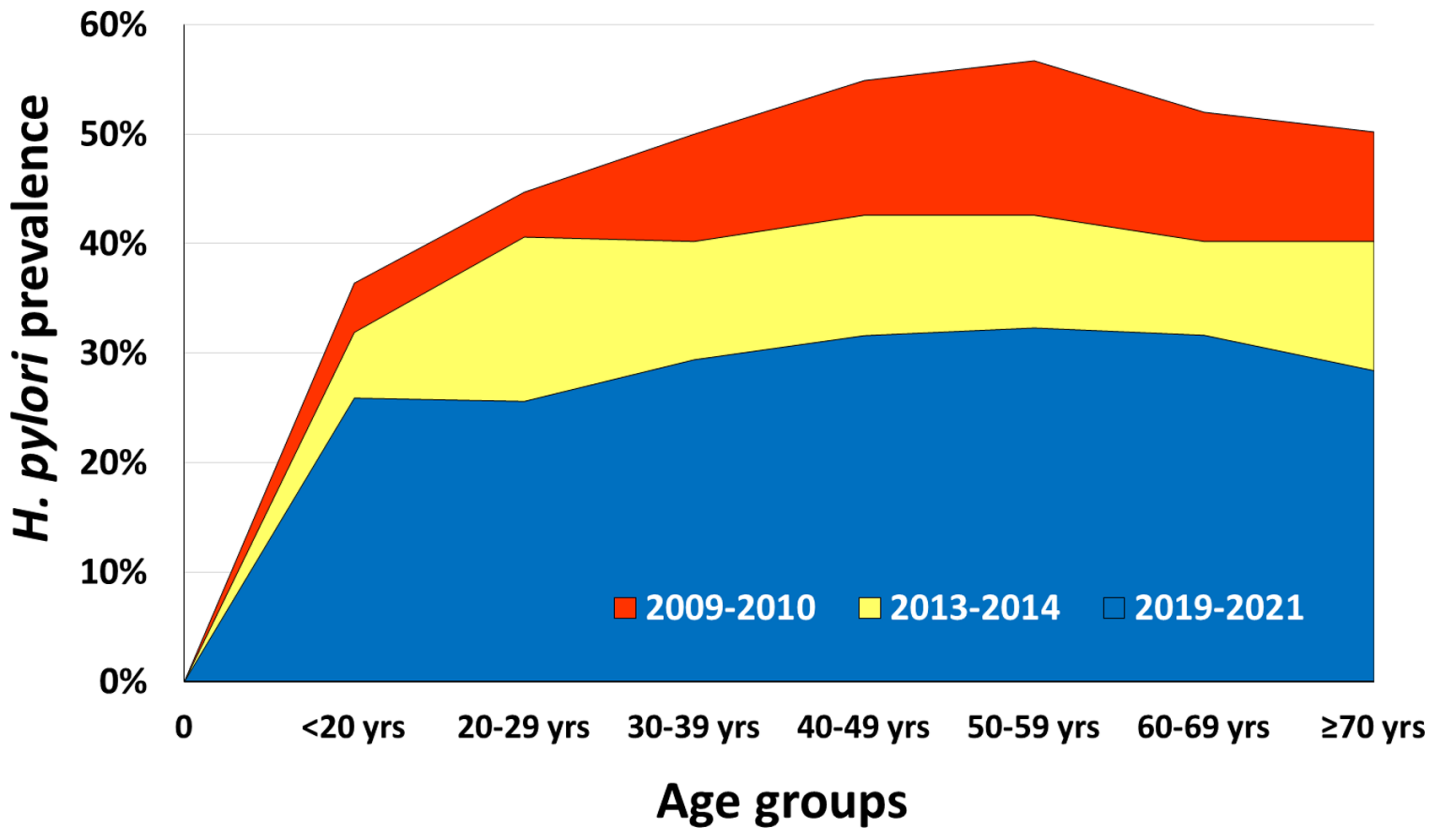
Age-specific *H. pylori* infection prevalence over time.

A similar decreasing trend in sex-specific and age-specific subgroups was simultaneously observed (Table 1). *H. pylori* prevalence in males was previously higher than that of females but the difference became insignificant in the recent period (OR = 1.05, 95% CI 0.99–1.12) (Figure 2). The age-specific *H. pylori* prevalence consistently increased before 40 years old, and always peaked at 50–59 years (Figure 3).

**Figure 4:**
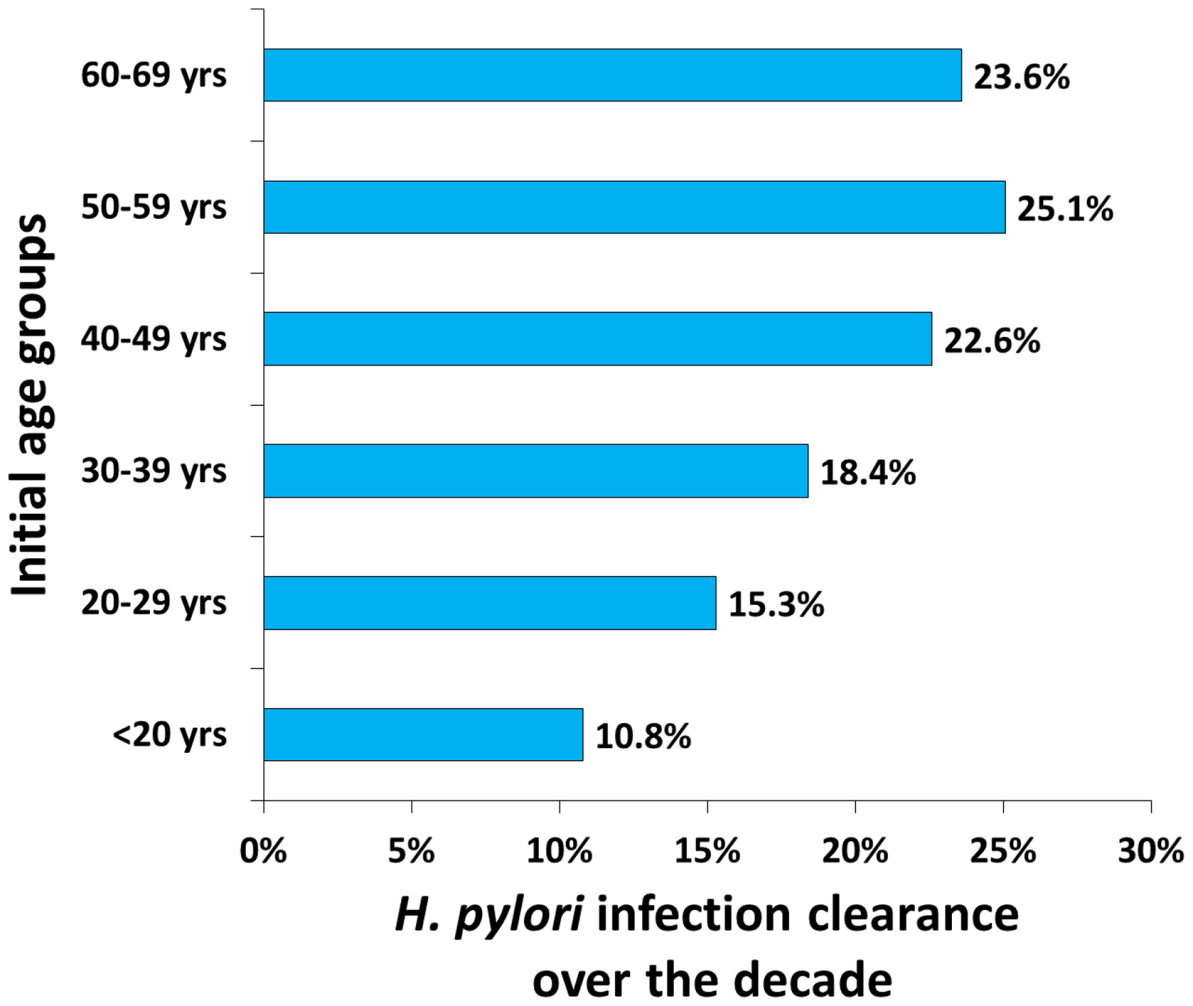
The estimated longitudinal clearance of each initial age group over the past decade.

Accordingly, the longitudinal clearance of *H. pylori* infection increased with age, and prevalence dropped to 22.6%, 25.1%, and 23.6% in the 40–49 years, 50–59 years, and 60–69 years initial age groups, respectively (Figure 4).

## Discussion

These findings confirm that the promotion of extensive *H. pylori* screening in health check-ups and eradication in general practice among urban health check-up populations can greatly contribute in decreasing the prevalence of *H. pylori* among any sex-specific or age-specific subpopulation in the long term. The 40–69-year-old age group has a heavier load of *H. pylori* infection, and therefore may have a stronger willingness to eradicate *H. pylori* (extrapolated due to greater clearance rate over the decade). Extensive UBT has a moderate predictive strength in gastric cancer screening and might be more cost-effective in the middle-aged subpopulation (40–59 years) (10).

Globally, a worldwide meta-analysis of 2,979,179 individuals from 71 countries or regions showed that the global overall prevalence of *H. pylori* infection decreased from 58.2% (1980–1990 period) to 43.1% (2011–2022 period), with a particularly sharp decline between 2011 and 2022 (11). Lower prevalence was associated with younger age, high-income countries, countries with a high level of health coverage, etc. (11). However, despite the dramatic decrease in our study, a meta-analysis of the domestic prevalence of *H. pylori* infection analyzed 670,572 participants from 26 provinces in China during the period 1983–2018, and found the overall prevalence slowly declined by only 0.9% per year (12). Another similar meta-analysis reported a decreased rate from 41.8% (2010–2014 period) to 40.0% (2015–2019 period) (13). A narrative comparison of the changes between worldwide and nationwide prevalence indicated that *H. pylori* infection control in China might be behind the global average efficacy. Therefore, the burden of *H. pylori* infection continues to be highly significant in China.

According to the recently released White Paper on *H. pylori* infection in China, the trend of overall prevalence declined gradually to below 50% between 1983 and 2018 (14). Furthermore, the prevalence was diverse among different subpopulations (between 35.4% and 66.4%), with higher rates in rural and adult subpopulations (14). The major reason for those changes was the rapid economic and societal development in China, as well as the obvious improvement in the public and medical health situation. Consequently, universal education, particular regarding health, increased the understanding and awareness of the importance of *H. pylori* screening and eradication. Updating the consensus on *H. pylori* eradication improved the capacity of the standardized and individualized management of *H. pylori* infection within the healthcare system. Crucial diagnostic tests were widely used and resistant strain detection techniques spread. The primary prevention of gastric cancer by population-based *H. pylori* eradication was piloted in many areas with a high incidence of gastric cancer.

Particularly, this improvement in *H. pylori* infection control may be attributed to the health policy of extending *H. pylori* screening and eradication in the Chinese population (15). The Chinese National Consensuses on the management of *H. pylori* infection were commonly updated following the serial Maastricht consensuses (16–18). The Third Chinese National Consensuses firstly accepted the eradication indication, including the asymptomatic but infected candidates. After the Kyoto global consensus (19), the Fifth Chinese National Consensuses recommended the eradication for all infected persons (5). Additionally, the Fifth Chinese National Consensuses emphasized health education and public awareness of gastric cancer control involving *H. pylori* screening and eradication. Therefore, these main changes improved *H. pylori* infection control in the Chengdu urban area over the past decade, and domestic updates in relevant health policy may be beneficial to gastric cancer prevention and control. In China in particular, it may be predicted that the inflammatory mechanism (i.e., *H. pylori*-associated atrophic gastritis) associated with gastric cancer would be controlled alone with the declining public prevalence of *H. pylori* (20).

Therefore, the routine screening of *H. pylori* was included in the health check-up of the urban population in West China Hospital for more than a decade, and infected persons were commonly recommended standardized intervention and surveillance. The major reason for the rapid decrease in our report could be the high-selected feature of urban observations. Education level, self-awareness of health management, and willingness to participate in *H. pylori* screening and eradication should be apparently higher among the observations than the average level of the common population. The imbalance between urban and rural areas limits the overall declining rate of *H. pylori* prevalence.

Wang, et al. used a Markov model to predict the incidence of and mortality from gastric cancer during the period 2021–2035 among Chinese people born between 1951 and 1980 (4). The various protocols of *H. pylori* eradication and endoscopic screening and surveillance were evaluated regarding their affordability and cost-effectiveness from the perspective of nationwide gastric cancer prevention. We believe primary and secondary prevention must be fairly important with regard to gastric cancer achieving the nationwide goal of an improvement of 5-year overall survival by 15% between 2016 and 2030 in China (10, 21). However, the low proportion of early gastric cancer (generally no more than 20%) is still a difficulty in improving population survival outcome of gastric cancer in China (22).

Some limitations of the present study need to be considered. First, this analysis included extracted data from two published studies, and thus, the relevant original data were unavailable to estimate the proportion of repeated observations within three periods. Second, the health check-up population in West China Hospital was almost entirely composed of staff from public institutions and urban enterprises. Therefore, it does not reflect the overall domestic prevalence situation. Last, the exact rates and details of eradication in separate cross-sections were unavailable, and therefore, no further understanding and evaluation of eradication could be obtained in this study.

In short, this research confirmed that the updated health policy of extending *H. pylori* screening and increasing eradication could greatly contribute in decreasing the long-term prevalence of *H. pylori* among urban health check-up populations.

## Data availability statement

The raw data supporting the conclusions of this article will be made available by the authors, without undue reservation.

## Ethics statement

The studies involving humans were approved by the study was approved by the Biomedical Ethical Committee of West China Hospital, Sichuan University (id: 2015-151-V2; 2018-215-V1). The studies were conducted in accordance with the local legislation and institutional requirements. Written informed consent for participation was not required from the participants or the participants' legal guardians/next of kin in accordance with the national legislation and institutional requirements.

## Author contributions

J-CZ and M-YW for the data collection and analysis. X-ZC and YH for the study conception and conduction. X-ZC for the writing. J-KH for the academic inspection. All authors contributed to the article and approved the submitted version.
